# Immune, molecular and genetic profiles of gastric signet ring cell carcinoma: Recent progress and future challenges

**DOI:** 10.1002/ijc.70416

**Published:** 2026-03-27

**Authors:** Qian Wang, Shuai Zhou, Xiongchao Fang, Xianli He, Gang Wang, Nan Wang

**Affiliations:** ^1^ Department of General Surgery, Tangdu Hospital Fourth Military Medical University Xi'an Shaanxi China

**Keywords:** CLDN18.2, CXCL13, diffuse gastric cancer, gastric signet ring cell carcinoma, therapeutic implications

## Abstract

Gastric signet ring cell carcinoma (GSRCC) is a special type of gastric cancer common in young women. Diffuse gastric cancer (DGC) begins with intramucosal lesions comprising differentiated GSRCC cells. Genetically, GSRCC and DGC are clonally identical, with their morphology influenced by extracellular Wnt signaling. Interestingly, Wnt activation facilitates the transition of indolent GSRCC cells into more invasive DGC cells, indicating the high plasticity of GSRCC cells. With respect to its cellular origin, GSRCC may originate from MUC5AC‐/MUC6‐ pre‐pit cells in the proliferative area of the gastric gland. Importantly, the tumor immune microenvironment (TIM) of GSRCC has unique characteristics. Compared with that of non‐GSRCC, the TIM of GSRCC seems to be in a relatively “quiescent” state, and CD4^+^ and CD8^+^ T cells are difficult to activate. Moreover, compared with non‐GSRCC patients, GSRCC patients have significantly greater Treg infiltration and significantly fewer CD8^+^ T effector cells. This immune “quiescent” state may explain the poor response to immunotherapy in patients with GSRCC. Notably, the depletion of CXCL13 derived from exhausted CD8^+^ T cells (CD8‐Tex) and the absence of mature tertiary lymphoid structures are key reasons for the low response to immunotherapy in patients with GSRCC. Therefore, for patients with GSRCC, enhancing the ability of CD8‐Tex cells to produce CXCL13 may be the key to improving the immune therapy response. In this review, we explore recent key findings on GSRCC, focusing on molecular mechanisms, immune regulation, and prospective research directions to improve clinical applications.

## Graphical abstract

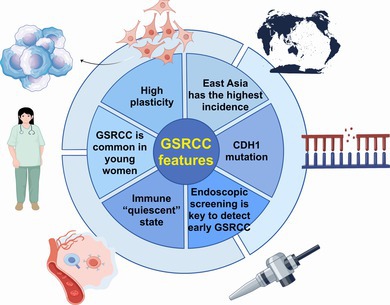

**Abbreviations**
CAR‐T: chimeric antigen receptor T‐cell
CD8‐Tex: exhausted CD8^+^ T cells
CLDN18.2: Claudin18.2
DGC: diffuse gastric cancer
ESD: endoscopic submucosal dissection
GPER: G protein‐coupled estrogen receptors
GSRCC: gastric signet ring cell carcinoma
HPA: heparinase
M/PDA: moderately/poorly differentiated adenocarcinoma
MSMB: Microsperin β
PCC‐NOS: poorly cohesive carcinoma not otherwise specified
SFRP2: secreted frizzled‐related protein 2
SHM: somatic hypermitation
Teff: CD8+T effector; Treg, regulatory T cells
TIM: tumor immune microenvironment
TLSs: tertiary lymphoid structures

## 1 INTRODUCTION

Gastric signet ring cell carcinoma (GSRCC) is a special type of gastric cancer that accounts for approximately 9.9% of all gastric cancers.^1^ It is characterized by the accumulation of a large amount of mucus inside the cell and compression of the nucleus to the edge in a “signet ring‐like” shape.^2^ It has a high degree of malignancy and strong invasiveness.^3^ There are certain differences in the incidence and epidemic trends of GSRCC worldwide.^4^ A Swedish study revealed that compared with local Swedish women, Southeast Asian women had a 6.7 times higher standardized incidence of GSRCC.^5^ In a study of 10,000 gastric specimens in Japan, GSRCC accounted for 10.2% of gastric cancer cases, indicating that the incidence rate in different regions may be affected by environmental, genetic and other factors.^6^

A study systematically revealed the fundamental differences in the tumor immune microenvironment between Asian and Western gastric cancer cohorts by comparing their tumor tissue samples. Research has revealed that tumors in non‐Asian patients exhibit significantly increased infiltration of cytotoxic T cells (CD8^+^ T cells) and memory T cells (CD45R0^+^ T cells), indicating a more active immune system response against the tumors. However, this stronger immune infiltration does not translate to a better prognosis. In contrast, tumors in Asian patients exhibit higher levels of immunosuppressive Treg infiltration. This study further revealed that the macrophage‐to‐T‐cell (CD68/CD3) ratio serves as a critical prognostic indicator, with this ratio typically being higher in non‐Asian patients and associated with poorer outcomes.^7^ These findings suggest that the biological behaviors of gastric cancer in Eastern and Western populations are regulated by distinct immune mechanisms, providing important insights for the development of region‐specific immunotherapy strategies in the future.

Patients with GSRCC have unique clinical features. This disease is more common in middle‐aged and young people, especially young women. Previous studies have shown that the occurrence of this disease may be related to vigorous estrogen metabolism in young women.^8^, ^9^ The clinical manifestations of GSRCC are similar to those of other types of gastric cancer, but the early symptoms are insidious, and most patients are diagnosed in moderate or advanced stages.^10^, ^11^ Typical manifestations include upper abdominal pain, weight loss, gastrointestinal bleeding, etc. In the late stage, abdominal masses, ascites, or distant metastasis symptoms may occur.^12^, ^13^

Early and accurate diagnosis of GSRCC is crucial for the treatment and prognosis of patients. Endoscopic examination is among the commonly used diagnostic methods.^1^ Early GSRCC often presents as pale and flat lesions under the microscope, without mucosal abnormalities such as ulcers, elevations, or depressions. Under magnifying endoscopy combined with narrow‐band imaging, irregularities in glands and microvessels can be observed, as well as a unique “stretching sign,” which refers to the elongation or dilation of the structure. This feature may aid in the early diagnosis of GSRCC.^14^ Notably, the tumor immune microenvironment of GSRCC seems to be in a relatively “quiescent” state, which may be one of the reasons for the poor response to immunotherapy in GSRCC. In this review, we explore the recent main findings on GSRCC, focusing on molecular mechanisms, immune regulation, and prospective research directions to improve clinical applications.

## 2 THE CELLULAR ORIGIN OF GSRC

The fundus glands, also known as acid‐secreting glands, are the most numerous and functionally crucial glands in the gastric mucosa.^15^ It is distributed mainly in the fundus and body of the stomach and presents as a branched tubular structure.^16^ The glands are successively divided into the isthmus, neck and base from the outside to the inside.^17^ All kinds of cells in the mucosal layer originate from pluripotent stem cells located in the isthmus of the gland. Pluripotent stem cells can differentiate into three main types of precursor cells: pre‐pit cells, pre‐parietal cells, and pre‐neck cells.^18^, ^19^ Pre‐pit cells without secretory granules in the isthmus migrate upwards and differentiate into pit cells that secrete mucus, while pre‐neck cells migrate downward and differentiate into cervical mucous cells and principal cells.^20^, ^21^ Pre‐parietal cells can migrate and differentiate into parietal cells in both upward and downward directions.^22^

**FIGURE 1:**
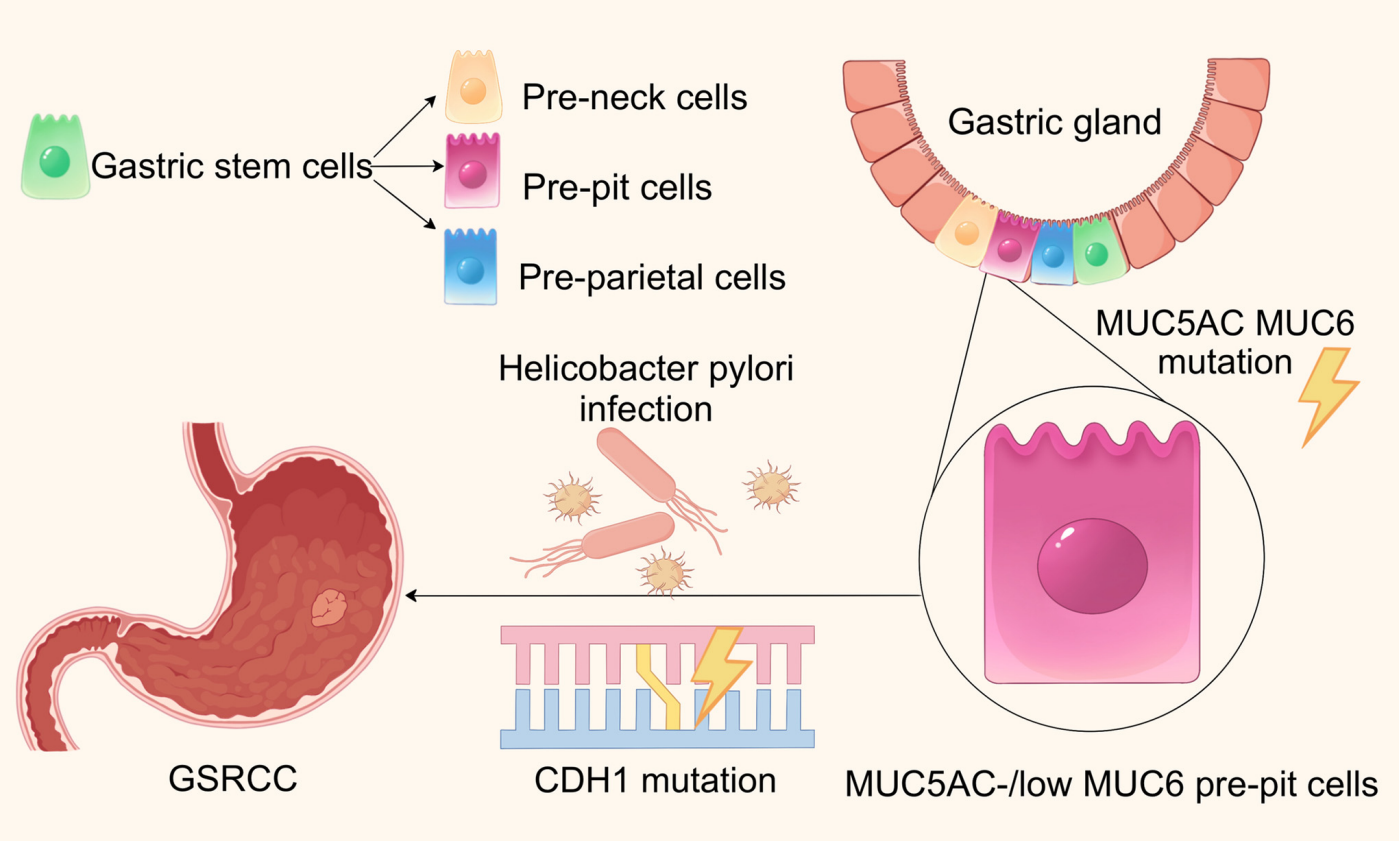
The cellular origin of GSRCC. GSRCC may originate from pre‐pit cells with low expression of MUC5AC/MUC6 in the proliferative area of the gastric gland, which exhibit dysplasia during the precancerous stage. MUC5AC‐/MUC6‐ pre‐pit cells gradually develop into GSRCC under the combined effects of an external *Helicobacter pylori*‐induced inflammatory microenvironment and internal CDH1 gene mutations. GSRCC, gastric signet ring cell carcinoma.

There are currently multiple hypotheses regarding the cellular origin of GSRCC. A study suggested that GSRCC might originate from pre‐pit cells with low expression of MUC5AC/MUC6 in the proliferative area of the gastric gland, which exhibit dysplasia during the precancerous stage (Figure 1).^23^ Furthermore, abnormal differentiation of pluripotent stem cells can produce signet ring cells, which may be associated with specific gene mutations such as *CDH1*.^24^ Moreover, *Helicobacter pylori* infection facilitates the malignant transformation of pluripotent stem cells through the induction of chronic inflammation and modification of the gastric mucosal microenvironment.^25^, ^26^ In future research, cell lineage tracing methods can be used to label MUC5AC‐/MUC6‐ pre‐pit cells or pluripotent stem cells in the glandular isthmus to further verify the validity of the above hypothesis.

## 3 MOLECULAR PATHWAYS REGULATING THE PLASTICITY OF GSRCC

**FIGURE 2:**
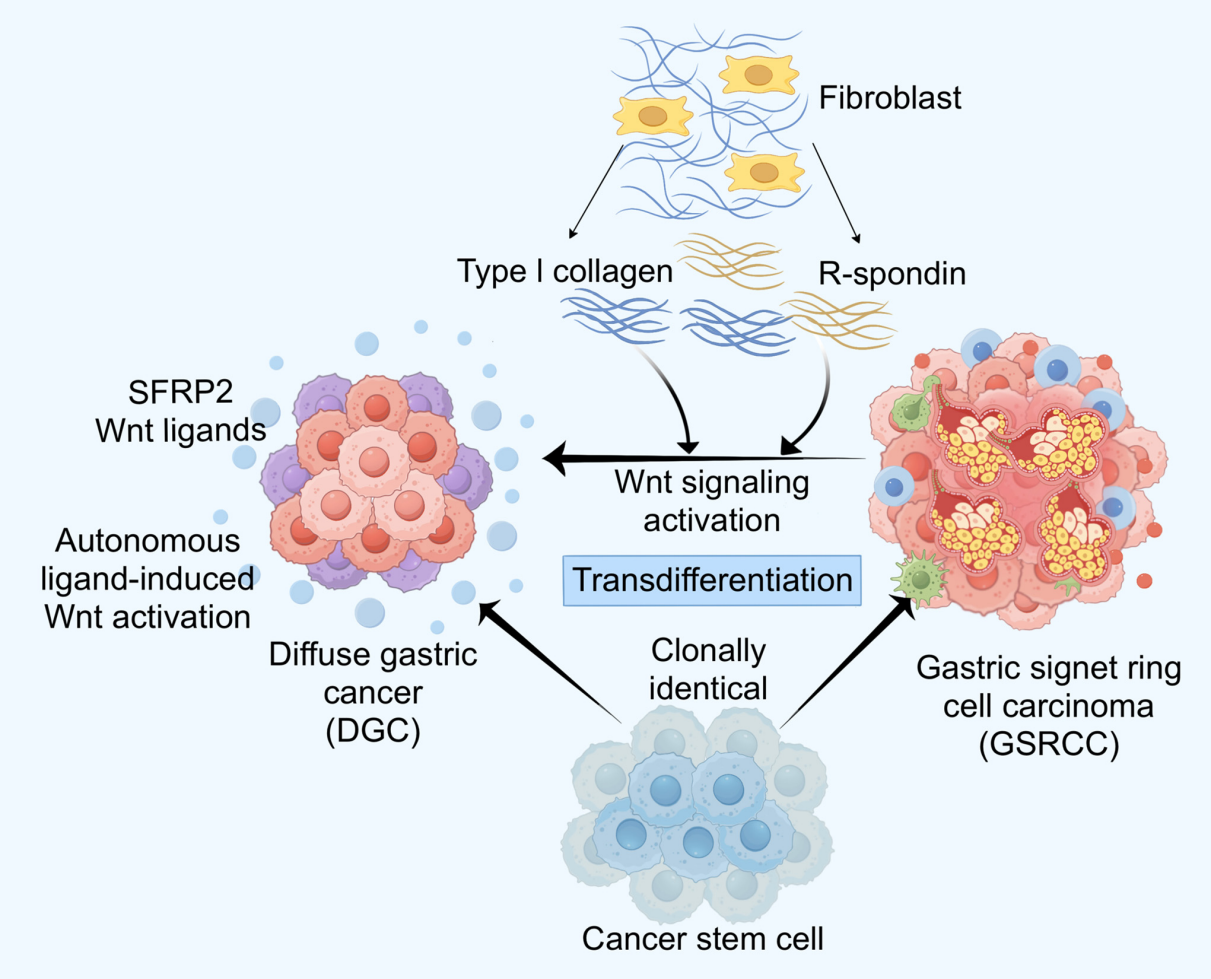
GSRCC has strong plasticity. Genetically, GSRCC and DGC are clonally identical, with their morphology influenced by Wnt signaling. Fibroblasts can activate the Wnt pathway by secreting type I collagen and R‐spondin. Moreover, DGC cells can autonomously activate the Wnt signaling pathway by upregulating the expression of Wnt ligands and secreted frizzled‐related protein 2 (SFRP2), thus enhancing ligand‐induced Wnt signaling. In conclusion, DGC‐derived ligand expression and extracellular matrix remodeling jointly maintain Wnt signaling and promote the histological transformation of GSRCC to DGC, indicating the high plasticity of GSRCC cells. DGC, diffuse gastric cancer; GSRCC, gastric signet ring cell carcinoma.

Diffuse gastric cancer (DGC) is currently classified as GSRCC and non‐GSRCC (i.e., poorly cohesive carcinoma not otherwise specified, PCC‐NOS).^27^ The development of DGC begins with intramucosal lesions and is composed mainly of differentiated nonproliferative signet ring cells.^24^ Togasaki et al. created a diffuse gastric cancer model by knocking out *CDH1* and/or *TP53* in normal human gastric organoids.^25^, ^26^ When Wnt and R‐spondin were removed from the culture medium, the DGC organoids transformed into GSRCC‐like structures. However, the activation of Wnt signaling prevents DGC from differentiating into GSRCC, confirming the association between Wnt signaling and histologic subtypes.^24^ Wnt signaling can keep DGC cells in an undifferentiated state when they disseminate away from the gastric cancer stem cell niche. In vivo experiments revealed that monoclonal organoids developed tumors with two histological regions, namely, GSRCC and DGC. These observations suggest that GSRCC and DGC are clonally identical, with their morphological features being modulated by the expression of extracellular Wnt and R‐spondin.^25^ Mechanically, DGC cells establish an autonomous source of Wnt pathway activation by upregulating the expression of Wnt ligands and “secreted frizzled‐related protein 2” (SFRP2), which enhances ligand‐induced Wnt signaling.^24^ Moreover, the deposition of type I collagen and R‐spondin secretion by fibroblasts effectively inhibited the differentiation of DGC cells. In conclusion, DGC‐derived ligand expression and extracellular matrix remodeling jointly maintain Wnt signaling and promote the histological transformation of GSRCC to DGC (Figure 2).^25^, ^26^, ^28^, ^29^ Therefore, understanding these mechanisms is highly important for the development of anticancer therapies targeting tumor plasticity.

## 4 IDENTIFICATION OF MOLECULAR MARKERS OF GSRCC

The identification of specific molecular markers for GSRCC is highly important for precise diagnosis and treatment. Currently, there is no absolute “specific” biomarker that exists only in GSRCC and not in other cancers. However, a series of highly characteristic and diagnostic biomarkers play crucial roles in pathological diagnosis and differential diagnosis. These markers are detected mainly in biopsy or surgical samples through immunohistochemical methods. The first most commonly used marker is cytokeratin (CK), such as CK7 and CK20. CK7 (+) and CK20 (+/−) are the most common immunophenotypes. Most GSRCC tissues express both CK7 and CK20 or only CK7. This combination helps distinguish it from colorectal signet‐ring cell carcinoma, which is typically CK7−/CK20+.^30^ The second most commonly used marker is E‐cadherin. Most GSRCCs have mutations or epigenetic silencing of the *CDH1* gene, resulting in the loss of expression of the E‐cadherin protein encoded by it. The absence of E‐cadherin is a pivotal factor contributing to the diffuse infiltration growth of tumor cells, as observed in the morphology of signet ring cells. Consequently, the loss of E‐cadherin expression is a distinctive alteration characteristic of GSRCC.^1^

Currently, there are no specific serum tumor markers for GSRCC. Common serum tumor markers for advanced GSRCC include CEA, CA19‐9, and CA72‐4. These markers are often elevated in patients with distant metastasis, such as in the liver or peritoneum. Their primary use is in monitoring treatment efficacy: a decrease in CEA levels posttreatment suggests effective therapy, whereas an increase indicates tumor recurrence or progression. Why are there no “specific” serum markers? The first reason is due to low tissue specificity. Markers such as CEA, CA19‐9, and CA72‐4 are produced by tumor cells and enter the bloodstream, but they are not unique to GSRCC. These antigens can also be found in various other cancers (e.g., colorectal, pancreatic, and lung) and some benign conditions (e.g., pancreatitis, cholangitis, and cirrhosis). Second, GSRCC is a type of diffuse gastric cancer that tends to infiltrate and grow within the gastric wall rather than form a lump protruding toward the lumen. This growth pattern may result in a later breakthrough of the basement membrane into blood vessels and lymphatic vessels, leading to delayed or insignificant release of serum markers. Consequently, early‐stage patients may have normal serum tumor marker levels.

The fibrinogen‐to‐albumin ratio (FAR) can be used to predict the prognosis of many malignant tumors.^31^, ^32^, ^33^ Recently, a retrospective cohort study of 330 patients with GSRCC demonstrated that the FAR‐CA125 score (FCS) is more accurate than the TNM stage in predicting the prognosis of resectable GSRCC.^34^ Kaplan–Meier analysis indicated that high FCS was associated with a poor overall survival rate. Moreover, the area under the receiver operating characteristic curve (AUC) of FCS (AUC = 0.724; *p* < 0.001; 95% CI, 0.669–0.779) was greater than that of CA125 (AUC = 0.673; *p* < 0.001; 95% CI, 0.613–0.734) and FAR (AUC = 0.662; *p* < 0.001; 95% CI, 0.601–0.722), suggesting that the developed FCS‐based nomogram may be an effective tool for helping clinicians determine treatment strategies. In addition, the derived monocyte‐to‐lymphocyte ratio (dMLR) may serve as a promising novel preoperative biomarker to predict lymph node metastasis in patients with GSRCC. The diagnostic sensitivity and specificity of the dMLR reached 60.3% and 72.2%, respectively.^35^ Similarly, a new systemic immune‐inflammation index (SII) based on peripheral lymphocytes, neutrophils, and platelets has been linked to poor prognosis in patients with GSRCC.^36^ A high SII was strongly linked to larger tumors, serosa infiltration, lymph node metastasis, and advanced TNM stage, all with *p* < 0.001. A low SII is associated with better clinical outcomes, providing a new prognostic tool for better treatment planning and stratification of patients.

Claudin18.2 (CLDN18.2) represents a promising therapeutic biomarker for GSRCC.^37^ A study from mainland China reported a positivity rate of CLDN18.2 in GSRCC as high as 95.2%,^38^ whereas another study from Japan reported a positivity rate of 55.2%.^39^ This discrepancy is likely primarily due to inconsistencies in detection methods and evaluation criteria.^40^ Recent findings indicate that CLDN18.2 may serve as a viable therapeutic target for GSRCC. In 2024, zolbetuximab was introduced globally as the first antibody to target CLDN18.2. Clinical studies have shown that compared with chemotherapy alone, zolbertuximab combined with chemotherapy can significantly prolong the overall survival of CLDN18.2‐positive gastric cancer patients. Nevertheless, the efficacy of zolbertuximab in GSRCC requires further validation through large‐scale clinical trials in the future. Furthermore, Her‐2 could serve as a biomarker for assessing the efficacy of trastuzumab in patients diagnosed with GSRCC.^41^ In addition, a study of 89 advanced GSRCC patients revealed a PD‐L1 expression rate of 40.4% and a PD‐1 expression rate of 18.0%, indicating that immune checkpoint inhibitors may have a potential role in the treatment of GSRCC, but these findings require further clinical validation.^42^

With the development of artificial intelligence and sequencing technology, more and more molecules, such as heparinase^43^ and microsperin β,^23^, ^44^, ^45^ have been found to be expressed at significantly higher levels in GSRCC than in other gastric cancers. This can facilitate the identification of cellular differentiation states and tumor heterogeneity within these tumors.

Anterior Gradient 2 (AGR2) is a protein disulfide isomerase that is overexpressed in various cancers, including GSRCC.^46^ Furthermore, AGR2 can be secreted by signet ring cell carcinoma cells and subsequently absorbed by fibroblasts, where it activates these cells and facilitates tumor progression. AGR2 can be easily detected in body fluids, providing noninvasive diagnostic potential, and serves as a potential therapeutic target since it is a tumor‐associated antigen that is not found in normal cells.^46^

In addition, through spatial mass spectrometry metabolomics technology, multiple lipid biomarkers related to GSRCC, such as phosphatidylethanolamine N‐methyl and phosphatidylethanolamine, have been identified, providing new insights for the study of its pathogenesis and therapeutic targets.^47^ However, this study has some limitations. The sample size is relatively small (*n* = 4). Further validation is needed to determine whether these metabolites can serve as specific diagnostic markers for GSRCC. Moreover, the role of these metabolites in the progression of GSRCC still needs further validation.

Another study compared the differences in miRNA expression between GSRCC and tubular adenocarcinoma and revealed 13 differentially expressed miRNAs, such as miR‐30a and miR‐26b.^48^ Furthermore, an analysis of samples from GSRCC and gastric adenocarcinoma was conducted utilizing Agilent microarray technology. The findings indicated that the expression of hsa‐miR‐665 and hsa‐miR‐95 was downregulated in GSRCC but upregulated in gastric adenocarcinoma.^49^ However, the use of these molecules as biomarkers, such as their specificity, sensitivity and feasibility in clinical practice, remains unclear and still needs to be further confirmed in future clinical research.

## 5 GENOTYPE–PHENOTYPE CORRELATIONS IN GSRCC

**FIGURE 3:**
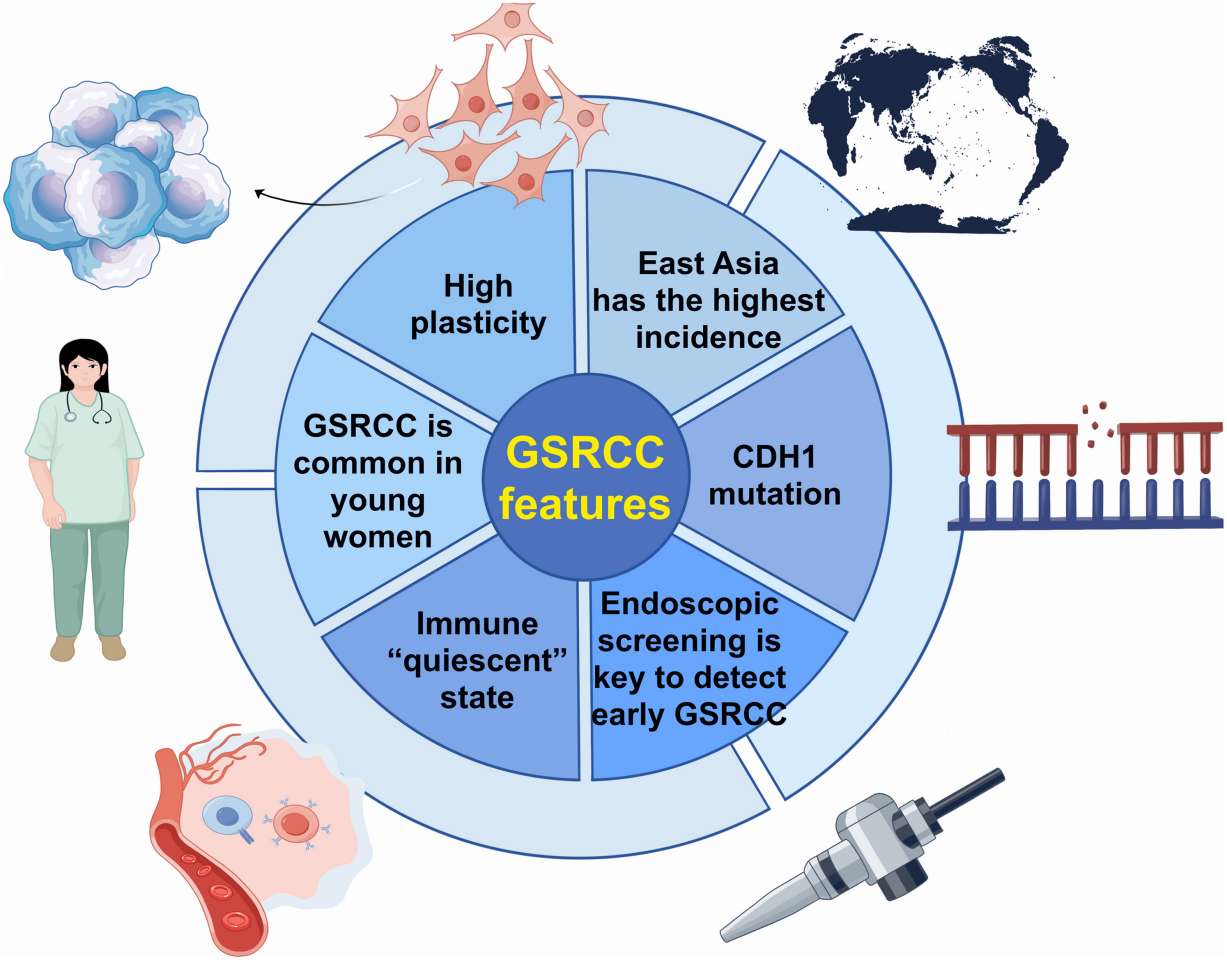
The incidence and clinical characteristics of GSRCC. GSRCC has the following characteristics: (1) it is more common among young women; (2) it has the highest global incidence rate in East Asia; (3) the CDH1 gene, which is responsible for encoding the E‐cadherin protein, is frequently mutated in GSRCC; (4) GSRCC has strong plasticity and can transform into diffuse gastric cancer under certain conditions; (5) the immune microenvironment of GSRCC is in a quiescent state; and (6) endoscopic screening is crucial for the early diagnosis and treatment of GSRCC. GSRCC, gastric signet ring cell carcinoma.

GSRCC has unique molecular biological features. *CDH1*, a tumor suppressor gene on chromosome 16q22, consists of 16 exons over 100 kb and encodes the E‐cadherin protein.^50^, ^51^ E‐cadherin plays a pivotal role in the establishment and maintenance of polarized and differentiated epithelial tissues throughout developmental processes.^52^ *CDH1* mutations can result in the loss of E‐cadherin expression, which subsequently impairs intercellular adhesion and facilitates tumor invasion and metastasis.^53^, ^54^ Since the first report in 1998,^55^ more than 100 germline *CDH1* mutations have been identified in hereditary diffuse gastric cancer (including signet ring cell carcinoma) (Figure 3). The mutations are mainly truncating mutations, often caused by frameshift mutations, single nucleotide variants or exon/intron splice site mutations.^55^, ^56^, ^57^ Overall, no specific “hot spots” have been identified, and the pathogenic variants are distributed throughout the entire gene. Furthermore, several reports have documented the presence of the same pathogenic variant in multiple unrelated families. Notable examples include the c.1003C>T variant in exon 7, the c.1901C>T variant in exon 12, the 1137G>A splicing mutation in exon 8, and the c.88C>A missense variant.^58^, ^59^, ^60^, ^61^ Truncating mutations are typically considered pathogenic, but the impact of missense mutations is unclear.^62^ Comprehensive family data and experimental data are needed to predict the pathogenicity of missense mutations. Without these data, the use of *CDH1* missense mutations to assess risk may be inappropriate.^63^

Significantly, the *CDH1* mutation rates significantly differ between Asian and Western cohorts. In Western countries with a low incidence of gastric cancer (e.g., Europe and North America), the detection rate of *CDH1* germline pathogenic variants reaches 30%–50% in families meeting clinical criteria for hereditary diffuse gastric cancer (HDGC).^64^ In contrast, in high‐incidence East Asian regions (e.g., Japan and Korea), even in those with a clear family history of gastric cancer, the *CDH1* mutation detection rate is only 7.4%, which is significantly lower than that in Western countries.^65^ This disparity suggests that the high incidence of gastric cancer in East Asian populations may be more strongly associated with environmental factors (such as *H. pylori* infection) and other genetic backgrounds (e.g., *ALDH2* allele deficiency). Furthermore, a meta‐analysis incorporating 44 studies revealed that *CDH1* rs16260 is significantly associated with an increased risk of HDGC in Caucasians. In contrast, the *CDH1* rs5030625 variant enhances HDGC susceptibility in Asian populations.^66^ These findings indicate substantial differences in *CDH1* gene polymorphisms for gastric cancer predisposition between Eastern and Western cohorts.

National Comprehensive Cancer Network (NCCN) guidelines recommend that individuals carrying pathogenic *CDH1* mutations should undergo prophylactic gastrectomy or routine endoscopic surveillance with multiple biopsies every 6–12 months, which can significantly reduce the risk of cancer.^59^, ^67^ With respect to the appropriate age for undergoing prophylactic gastrectomy, the NCCN generally advises that the procedure should not be performed before the age of 18. In contrast, the International Gastric Cancer Linkage Consortium (IGCLC) recommends the earliest optimal age for this procedure to be 20 years. Additionally, Tan et al. and Moreira et al. shared similar views with the IGCLC.^68^, ^69^ Despite differing age recommendations by the NCCN and IGCLC, the international consensus is that prophylactic gastrectomy should not be performed on individuals younger than 18 years. However, van der Post et al. recommend that individuals should take into account their personal circumstances and adopt a personalized approach when determining the appropriate timing for prophylactic gastrectomy.^53^ For example, in a report by Wickremeratne et al.,^70^ a 16‐year‐old asymptomatic carrier of a *CDH1* mutation underwent total gastrectomy, despite having two normal gastroscopy results before gastrectomy. The patient's family history included the deaths of the mother and aunt from gastric cancer at ages 39 and 21. This case represents the youngest *CDH1* carrier who underwent prophylactic gastrectomy, which was performed years earlier than the guidelines typically recommend.

In addition to *CDH1* mutations, *CTNNA1* mutations are also common in GSRCC.^71^, ^72^ The *CTNNA1* gene is located on chromosome 5q31 and encodes α‐1 catenin, a protein that plays a crucial role in intercellular adhesion. Loss‐of‐function mutations in *CTNNA1* impair E‐cadherin‐mediated cell adhesion, causing cells to become loosely connected and invasive, which is an early step in the development of GSRCC.^73^ Utilizing molecular, clinical, and population data from 1308 individuals in 351 *CTNNA1*‐variant carrier families and 37,428 noncarriers of European and American ancestries, Lobo et al. reported that compared with individuals having the wild‐type gene sequence, *CTNNA1* truncating mutation carriers have a 7‐fold increased risk of gastric cancer, whereas those with *CDH1* truncating mutations face a 38‐fold increased risk.^74^ *CTNNA1* mutations pose a slightly lower gastric cancer risk than *CDH1* mutations but remain clinically significant. Notably, not all *CTNNA1* variations are clinically significant. Only loss‐of‐function mutations (such as truncating mutations, large deletions, or splice site mutations) are clearly associated with GSRCC. Most missense variations are classified as of uncertain significance unless further functional experimental evidence supports their pathogenicity. In some cases of GSRC, *TP53* gene mutations are associated with pathological characteristics such as tumor invasion depth and lymph node metastasis.^75^ Whole‐genome sequencing of GSRCC revealed that CLDN18‐ARHGAP26/6 fusion occurred frequently, and patients carrying this fusion had poor survival outcomes and were insensitive to oxaliplatin/fluoropyrimidine chemotherapy.^76^ Recent studies have shown that other genes, such as *ARID1A*, *PIK3CA*, *BAP1*, *p16*, and *ERBB2*, may also be mutated in GSRCC, which may affect multiple biological processes, such as cell proliferation, apoptosis, and metabolism.^71^, ^77^ Although these mutations are relatively rare and the sample sizes in some existing studies are too small to draw any conclusions, additional research is warranted to investigate the association between GSRCC and other potential candidate genes beyond *CDH1*.^71^, ^72^

## 6 MOLECULAR PATHWAYS SHAPING THE GSRCC IMMUNE LANDSCAPE

**FIGURE 4:**
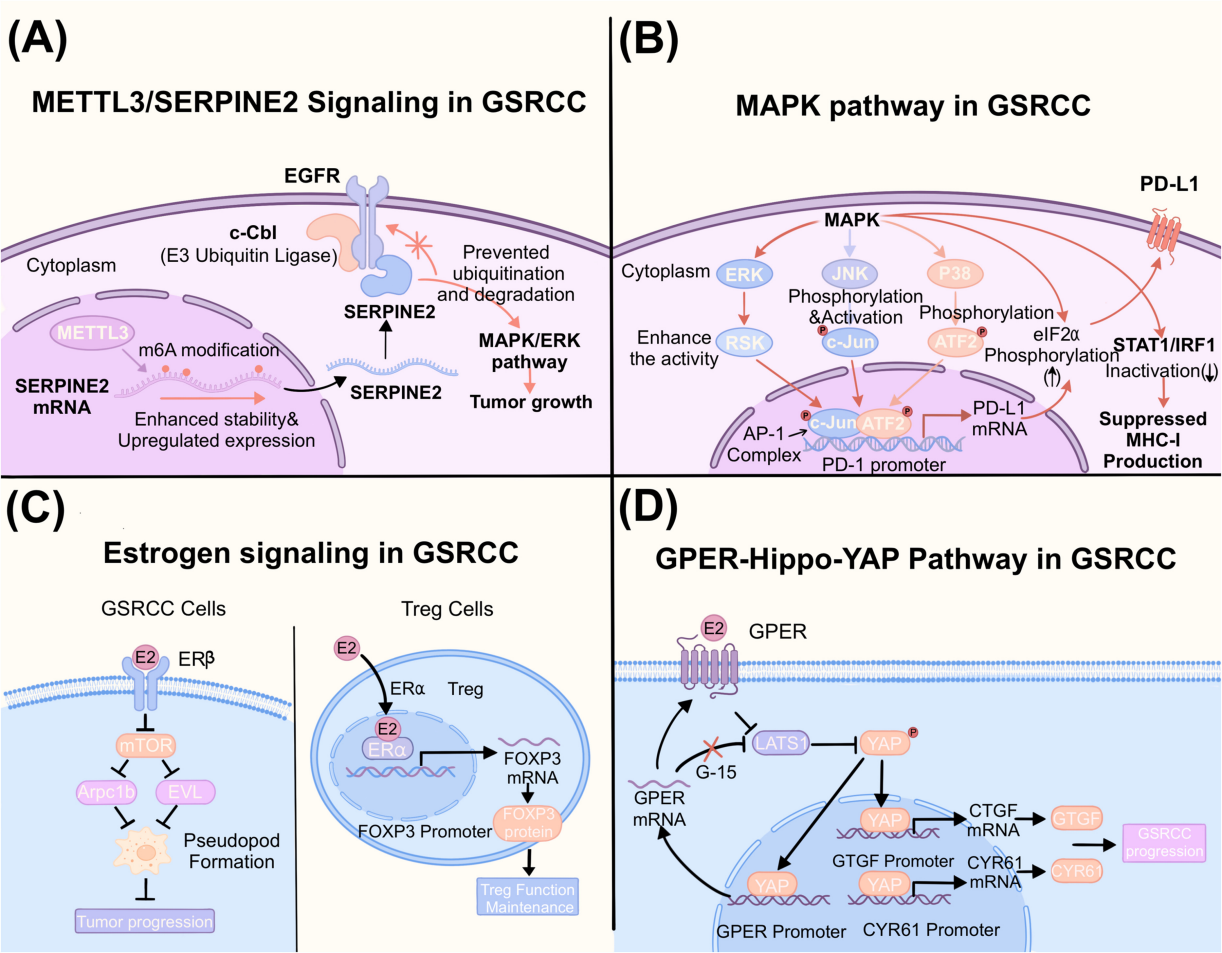
Molecular pathways related to GSRCC. (A) METTL3 enhances the stability of SERPINE2 mRNA via m^6^A modification and induces the upregulation of SERPINE2 expression.^79^ SERPINE2 competes with the E3 ubiquitin ligase c‐Cbl for binding to EGFR, thus preventing the ubiquitination‐mediated degradation of EGFR, activating the downstream MAPK/ERK pathway, and promoting tumor growth.^80^ (B) MAPK pathway (ERK, JNK, p38) increase the transcriptional activity of AP‐1, enabling its efficient binding to the promoter region of the PD‐L1 gene to initiate transcription.^23^, ^81^, ^82^, ^83^, ^84^, ^85^ In addition, the activation of the MAPK pathway resulted in the suppression of MHC‐I production via the inactivation of the STAT1/IRF1 signaling pathway while concurrently facilitating the translation of PD‐L1 by increasing the phosphorylation of eIF2α.^86^ (C) ERβ can inhibit pseudopodia formation through the mTOR‐Arpc1b/EVL signaling pathway to suppress the progression of GSRCC.^87^ ERα signaling plays a crucial role in maintaining FOXP3 expression and the functionality of Treg cells through the direct interaction of ERα with the FOXP3 promoter.^88^ (D) GPERs inhibit LATS1‐mediated YAP phosphorylation and promote the nuclear translocation of YAP and activates the transcription of downstream oncogenes (e.g., CTGF and CYR61), driving the malignant progression of GSRCC.^89^ ERα, Estradiol‐mediated estrogen receptor α; ERβ, Estrogen receptor β; GPERs, G protein‐coupled estrogen receptors; GSRCC, gastric signet ring cell carcinoma.

Studies have shown that GSRCC has a unique immunosuppressive microenvironment.^78^ Compared with moderately/poorly differentiated adenocarcinoma (M/PDA), the B‐cell subclusters in GSRCC have distinctive properties, characterized mainly by the infiltration of less effective follicular B cells. Their performance in antibody production and immune responses is relatively weak, undermining antitumor immunity.^23^ Furthermore, a recent study demonstrated that METTL3/SERPINE2 signaling plays a central role in M2 tumor‐associated macrophage polarization in GSRCC. Mechanistically, METTL3 enhances the stability of SERPINE2 mRNA via m^6^A modification and induces the upregulation of SERPINE2 expression.^79^ (Figure 4) SERPINE2 competes with the E3 ubiquitin ligase c‐Cbl for binding to EGFR, thus preventing the ubiquitination‐mediated degradation of EGFR, activating the downstream MAPK/ERK pathway, and promoting tumor growth.^80^

In addition, a study using single‐cell sequencing revealed significant enrichment of the MAPK pathway (ERK, JNK, p38) in GSRCC, which plays a critical role in facilitating tumor evasion from T‐cell immunosurveillance.^23^ Mechanistically, the activation of the MAPK pathway resulted in the suppression of MHC‐I production via the inactivation of the STAT1/IRF1 signaling pathway while concurrently facilitating the translation of PD‐L1 by increasing the phosphorylation of eIF2α.^86^ The aberrantly activated MAPK pathway promotes the upregulation of PD‐L1 expression through multilevel regulation of the AP‐1 transcription factor complex.^81^ Specifically, the JNK pathway, as the predominant regulator, directly phosphorylates and activates c‐Jun, the core component of AP‐1.^82^ The p38 pathway primarily phosphorylates ATF2, another member of AP‐1, which forms a dimer with c‐Jun to enhance its transcriptional function.^83^ Moreover, the ERK pathway not only potentiates c‐Jun activity via downstream effectors (e.g., RSK) but also exhibits extensive crosstalk with both the JNK and p38 pathways.^84^, ^85^ Collectively, these three pathways synergistically increase the transcriptional activity of AP‐1, enabling its efficient binding to the promoter region of the PD‐L1 gene to initiate transcription.^81^ The activation of MAPK may be one of the main reasons for the formation of the “immunologically cold” microenvironment in GSRCC and its poor response to immunotherapy.

The single‐cell TCR and BCR sequencing results of another study revealed that the clonal expansion ability and TCR sharing ability of CD4+ and CD8+ T cells were significantly inhibited in GSRCC.^23^ Moreover, the weakened differentiation ability of IgG‐type plasma cells in GSRCC and the decreased somatic hypermutation (SHM) of BCR suggest the reduced immune activity of B cells and their affinity for tumor antigens.^23^ These results indicate that the immune “silent” state of GSRCC may be partially caused by the inhibition of T‐ and B‐cell clone expansion and obstruction of cell differentiation/transformation. However, this study has some limitations.^23^ The sample size is relatively small (*n* = 13), and analytical methods for multiomics are lacking.

**FIGURE 5:**
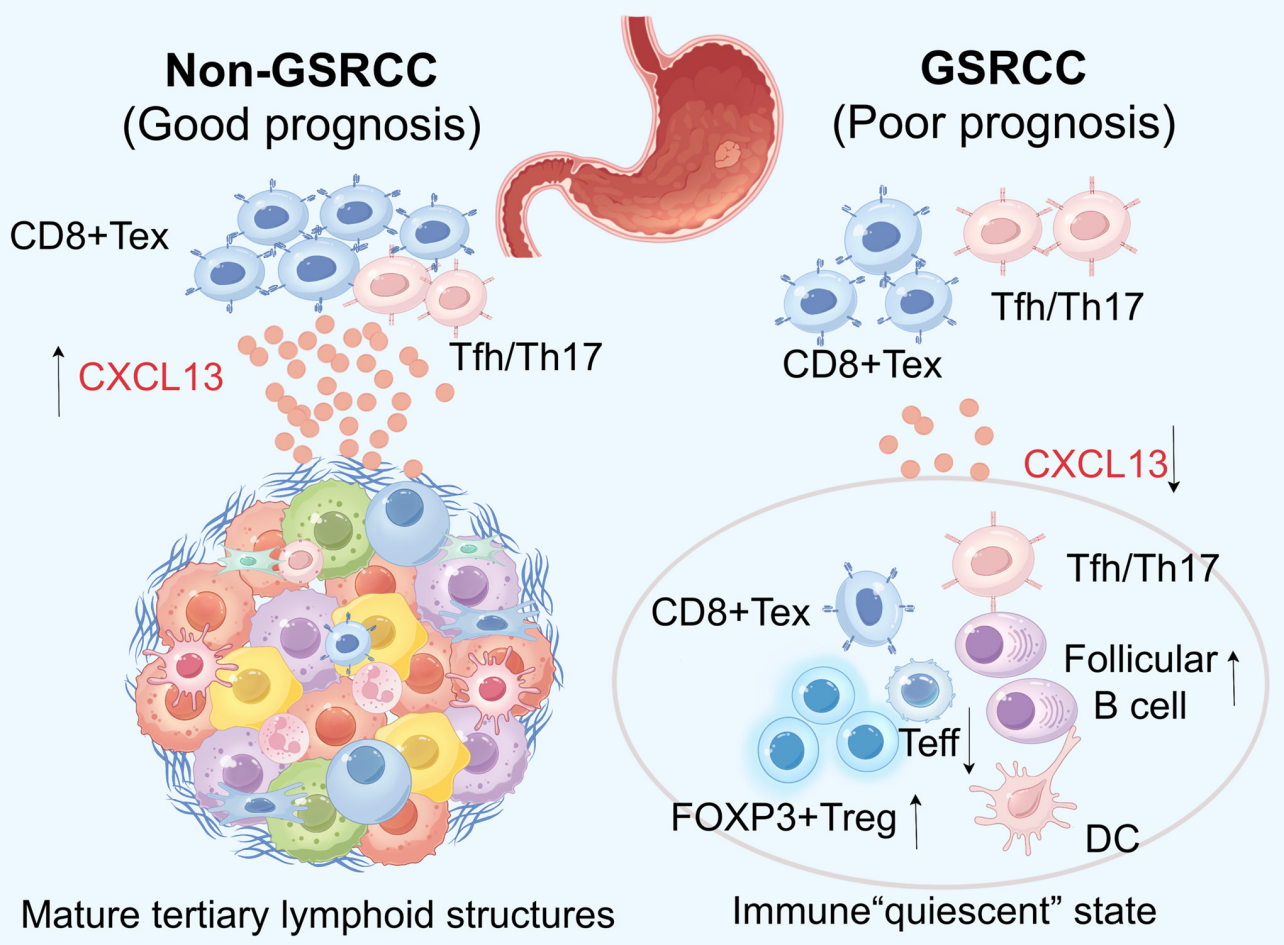
The tumor immune microenvironment (TIM) of GSRCC. Compared with that of non‐GSRCC, the TIM of GSRCC seems to be in a relatively “quiescent” state. Moreover, compared with non‐GSRCC patients, GSRCC patients had significantly higher FoxP3+ Treg infiltration and significantly fewer CD8+ T effector cells (Teffs). Notably, the depletion of CXCL13 derived from CD8+ T cells (CD8‐Tex) and the absence of mature tertiary lymphoid structures are key reasons for the low response to immunotherapy in GSRCC. GSRCC, gastric signet ring cell carcinoma.

CXCL13 predominantly originates from follicular helper T cells (Tfh), type 17 T helper cells (Th17), and exhausted CD8^+^ T cells (CD8‐Tex).^78^ CXCL13 plays a key role in maintaining immune responses in follicular germinal centers and normal humoral immune function. CXCL13 has been further proven to be a typical molecular marker for the formation of tertiary lymphoid structures (TLSs).^90^, ^91^, ^92^ Compared with that in non‐GSRCC cells, the expression level of CXCL13 in Tfh/Th17 cells and CD8‐Tex cells in GSRCC was significantly lower. In addition, many TLSs were assembled from T and B cells in non‐GSRCC, whereas almost no immune cell aggregation foci were observed in GSRCC. Moreover, the number and intensity of immune cell interactions were significantly reduced in GSRCC.^78^ In summary, the depletion of CXCL13 derived from CD8‐Tex cells and the absence of mature TLSs are key reasons for the low response of patients with GSRCC to immunotherapy^78^ (Figure 5). However, this study was mainly observational and had a small sample size (*n* = 7); thus, functional assays are needed to better understand the role of CXCL13 in immunosuppression in patients with GSRCC.^78^

scRNA sequencing revealed significant activation of estrogen response signaling in GSRCC. Previous studies have shown that young women are prone to GSRCC, and more than 80% of GSRCC cells can secrete mucin and express estrogen receptors, making them more likely to metastasize to the ovaries.^93^, ^94^ Furthermore, members of the heat shock protein family, such as HSP70, HSP90, and FKBP5, have been reported to activate the estrogen signaling pathway, which might promote the progression of GSRCC.^95^, ^96^, ^97^, ^98^ Conversely, estrogen receptor β can inhibit pseudopodia formation through the mTOR‐Arpc1b/EVL signaling pathway to suppress the progression of GSRCC.^87^ These findings indicate that different subtypes of estrogen receptors may play different roles in GSRCC.

Moreover, a higher percentage of Tregs is correlated with faster gastric cancer progression and reduced survival.^99^ Estradiol‐mediated estrogen receptor α (ERα) signaling plays a crucial role in maintaining FOXP3 expression and the functionality of Treg cells through the direct interaction of ERα with the FOXP3 promoter in human cervical cancer.^88^ Moreover, inhibition of ERα signaling can lead to a reduction in Tregs and is associated with an enhanced response to hormonal therapy in patients with breast cancer.^100^ Recent studies have confirmed that both Tregs and the ERα pathway are significantly elevated in GSRCC.^101^ Therefore, we speculate that the ERα pathway may also promote Treg infiltration in GSRCC, although this requires further experimental validation.

Interestingly, another study demonstrated that G protein‐coupled estrogen receptors (GPERs) inhibit LATS1‐mediated YAP phosphorylation by competitively binding to ARRB2, which promotes the nuclear translocation of YAP and activates the transcription of downstream oncogenes (e.g., CTGF and CYR61), driving the malignant progression of GSRCC.^89^ Moreover, YAP can directly bind to the *GPER* promoter to form a positive feedback loop, and the GPER inhibitor G‐15 can effectively block this loop and inhibit tumor growth. Therefore, targeting the GPER‐Hippo pathway may become a new strategy for overcoming drug resistance in the treatment of GSRCC.^89^

## 7 TREATMENT OF GSRCC

The treatment strategy for GSRCC varies depending on the patient's condition (Figure S1, Supporting Information). For early‐stage GSRCC, some cases can be treated by endoscopic submucosal dissection (ESD).^102^, ^103^ Surgical resection is another important treatment option for early GSRCC. The prognosis of patients after surgery is relatively good, and the incidence of lymph node metastasis is relatively low.^104^ For patients with advanced GSRCC, radical surgery combined with lymph node dissection is an important treatment approach, but the impact of different surgical scopes on patient survival is controversial. A study analyzing the National Cancer Database in the United States revealed that for patients with GSRCC, the overall survival rate of those who underwent partial gastrectomy was better than that of those who underwent total gastrectomy, suggesting that the choice of surgical approach should consider multiple factors comprehensively.^105^ Chemotherapy plays an important role in the treatment of GSRCC. The TEFOX regimen (docetaxel‐5FU‐oxaliplatin) has shown good efficacy as a first‐line treatment of advanced GSRCC, with an objective response rate of 66.1% and a disease control rate of 87.6%. Some initially unresectable patients can undergo secondary resection or radiotherapy after treatment with this regimen.^106^ In addition, a study analyzed 107 patients with locally advanced gastric cancer and revealed that tumors containing signet ring cell components had a relatively poor response to preoperative radiotherapy and chemotherapy. The higher the proportion of signet ring cells, the lower the probability of pathological complete response.^107^ Overall, GSRCC treatment should be personalized, considering the patient's specific situation, and the pros and cons of various strategies should be evaluated.

A clinical study suggested that the combination of MDM2 inhibitors and G2/M checkpoint inhibitors, including WEE1 or CHK1 inhibitors, can trigger a synergistic antitumor response in GSRCC cells by inducing DNA damage, providing a promising combined treatment approach for GSRCC.^108^ Another study revealed that a single high dose of vitamin C (HDVC) can induce selective growth inhibition and cell death in human GSRCC‐derived NUGC‐4 cells. These effects may be regulated by intracellular iron ions and/or intracellular oxidative stress levels.^109^

Since Pauling and Cameron first reported that HDVC could extend the survival of advanced cancer patients,^110^ its effectiveness has been debated for nearly 50 years. *The New England Journal of Medicine* refuted the therapeutic efficacy of HDVC in the treatment of cancer during the period from 1979 to 1985.^111^, ^112^ In 2010, HDVC attracted the attention of oncologists again because of detailed research on the pharmacokinetics and anticancer effects of vitamin C, outlining the best dose, administration method, and clinical protocol for its use in cancer treatment.^113^, ^114^ Although some trials still reported that HDVC was ineffective for cancer treatment,^115^, ^116^ more studies reported positive results when it was combined with radiotherapy, chemotherapy, or targeted therapy in advanced cancer patients. For instance, five clinical trials investigating the combination of HDVC with radiotherapy included 250 patients diagnosed with breast cancer, pancreatic cancer, malignant glioma, and bone metastases. Notably, the administration of HDVC resulted in a significant reduction of approximately 50% in the overall intensity score of patient symptoms during the treatment period.^117^, ^118^, ^119^, ^120^, ^121^ In addition to radiotherapy, another key strategy is combining HDVC with chemotherapy. A small number of studies have demonstrated no significant effect of HDVC in combination with chemotherapy; however, these trials were conducted with vitamin C concentrations below 1–1.5 g/kg/day and were predominantly completed prior to 2010.^122^, ^123^

After 2010, most trials used intravenous HDVC treatment with chemotherapy, achieving a 1–3‐fold increase. For example, in a randomized, open‐label, multicenter, phase III clinical trial, the efficacy of FOLFOX ± bevacizumab with or without HDVC was evaluated in patients with unresectable, untreated metastatic colorectal cancer. Patients harboring *RAS* gene mutations showed significantly improved progression‐free survival when treated with a combination of chemotherapy and HDVC compared with those who received chemotherapy alone.^124^ Another clinical trial examined the combination of HDVC with mFOLFOX6/FOLFIRI for advanced gastrointestinal cancer. Vitamin C was administered intravenously at a dosage of 1–1.5 g/kg/day, with a frequency of 2–3 times per week. The results demonstrated that patients with either wild‐type *RAS/BRAF* or mutated *KRAS* or *BRAF* cancers exhibited a favorable response to treatment.^125^ The above studies did not focus specifically on GSRCC, but they provided safety evidence for the combined application of high‐dose ascorbic acid and chemotherapy drugs in GSRCC.

With an enhanced understanding of the molecular mechanisms underlying tumorigenesis, targeted therapy has emerged as a promising treatment.^38^ The development of targeted drugs aimed at specific gene mutations or aberrant protein expression has provided new therapeutic options for certain patient populations. For HER‐2‐positive GSRCC, anti‐HER‐2 treatment is somewhat effective, but further screening is necessary.^126^, ^127^ Studies have shown that GSRCC patients with CLDN18‐ARHGAP26/6 fusion do not benefit from oxaliplatin/fluoropyrimidine chemotherapy, indicating that new targeted treatment strategies may need to be explored for this type of fusion.^76^ For patients with hereditary GSRCC carrying *CDH1* gene mutations, prophylactic gastrectomy is an option that can reduce the risk of developing the disease.^76^, ^128^, ^129^

The immune microenvironment of GSRCC has distinct features that are different from those of other types of gastric cancer.^78^ Compared with those of non‐GSRCC, the immune microenvironment of GSRCC seems to be in a relatively quiescent state, and CD4^+^ and CD8^+^ T cells are difficult to activate, thus affecting the normal function of B cells.^78^ In addition, the infiltration of immune cells is related to the prognosis of GSRCC. In patients with GSRCC, low CD3^+^ T‐cell infiltration is associated with poor overall survival.^42^ GSRCC exhibits a poor response to immune checkpoint inhibitors, primarily because of its unique biological characteristics that collectively create a highly immunosuppressive “cold tumor” microenvironment.^78^ Although some tumors may express PD‐L1, this biomarker has limited predictive value in this cancer type. The main contributing factors include the following: a diffuse growth pattern of tumor cells accompanied by a dense fibrous stroma, forming a physical barrier that prevents T‐cell infiltration and results in an immune desert state^130^; frequent downregulation of MHC class I molecule expression by cancer cells, leading to defective antigen presentation and impaired T‐cell recognition^78^; generally stable genomes with low tumor mutational burden and few neoantigens^23^; and classification as the “genomically stable” molecular subtype, which is least responsive to immunotherapy.^131^ Therefore, even when the immune “brake” is released using PD‐L1/PD‐1 inhibitors, the lack of sufficient and functionally competent T cells makes it difficult to achieve efficacy. Current treatment strategies are shifting toward exploring combination therapies that combine immunotherapy with chemotherapy, antiangiogenic therapy, and other approaches capable of altering the tumor microenvironment.

Novel immunotherapy technology has resulted in new hope for the treatment of GSRCC.^132^, ^133^ Chimeric antigen receptor T‐cell (CAR‐T) therapy enhances the ability to kill tumor cells through the genetic engineering of T cells to enable them to specifically recognize tumor antigens.^134^, ^135^, ^136^, ^137^ Currently, CAR‐T cells designed to target various tumor‐associated antigens are undergoing development. However, GSRCC is a highly invasive gastric cancer subtype that poses challenges for CAR‐T‐cell therapy because of its high heterogeneity and uneven or absent expression of target antigens on tumor cells, hindering the ability of CAR‐T cells to recognize all cancer cells.^138^ Moreover, GSRCC has a dense tumor microenvironment that is rich in stromal components such as fibroblasts and collagen fibers, forming a physical barrier that hinders CAR‐T‐cell infiltration. Furthermore, GSRCC has a strong immunosuppressive microenvironment, filled with regulatory T cells, tumor‐associated macrophages, and myeloid‐derived suppressor cells, and high expression of immunosuppressive factors such as TGF‐β and IL‐10, which can inhibit the activity and persistence of CAR‐T cells. Finally, ideal specific targets for GSRCC are lacking. The ideal target should be highly expressed in tumor cells and not expressed in normal tissues to avoid off‐target toxicity. This is difficult to find in GSRCC.^138^

Despite these significant challenges, researchers continue to explore this topic.^139^ Currently, CAR‐T‐cell clinical trials for gastric cancer (including GSRCC) focus mainly on Claudin 18.2 (CLDN18.2), which is highly expressed in most advanced GSRCCs.^38^ In a single‐arm, open‐label, phase 1 trial, CT041, an autologous CLDN18.2‐specific chimeric antigen receptor (CAR) T‐cell therapy, has shown good clinical results in patients with CLDN18.2‐positive advanced gastrointestinal cancers (including GSRCC). The overall response rate and disease control rate among all 98 patients were 38.8 and 91.8%, respectively.^140^ Similarly, in a randomized, open‐label, phase 2 trial, CT041 also demonstrated promising clinical results in patients with CLDN18.2‐positive advanced gastric or gastroesophageal junction cancer (including GSRCC) who were refractory to at least two previous lines of treatment. The administration of CT041 led to a significant improvement in progression‐free survival, with a duration of 9.07 months observed in the CT041 cohort compared with 3.45 months in the control cohort, while a manageable safety profile was maintained.^141^

In addition to CLDN18.2, CAR‐T‐cell studies targeting other tumor antigens, such as EpCAM, CEA, and CDH17, in gastric cancer (including GSRCC) are still ongoing (ClinicalTrials.gov identifiers NCT03563326, NCT06010862, NCT07179692, and NCT06937567). In conclusion, CAR‐T‐cell therapy for the treatment of GSRCC is still in the early stage of clinical exploration and is far from becoming the standard treatment. CLDN18.2 CAR‐T‐cell therapy (such as CT041) is currently the most promising direction, showing substantial efficacy and providing new hope for specific patient populations.

However, owing to the distinct challenges associated with GSRCC, such as high heterogeneity and strong immune suppression, the efficacy of simple CAR‐T‐cell infusion may be limited. In the future, the combination of CAR‐T cells with various strategies, such as chemotherapy, immune checkpoint inhibitors, and tumor vaccines, may have synergistic effects and improve treatment efficacy, which will become a research focus. Moreover, technologies such as proteomics and single‐cell sequencing should be used to discover more specific surface antigens for GSRCC, providing the possibility of finding better targets.

CAR structural optimization is also an important direction for technological innovation; for example, the use of bispecific CARs (such as CLDN18.2/HER2 bispecific CARs) can improve the specificity and efficacy of CAR‐T cells.^142^ The application of gene editing technology can further optimize the function of CAR‐T cells, for example, by using CRISPR‐Cas9 to knock out the PD‐1 gene in CAR‐T cells and enhance their antitumor activity.^143^ In addition, the preparation process of CAR‐T cells is constantly being optimized; for example, induced pluripotent stem cells (iPSCs) are being used to prepare universal CAR‐T cells, reducing treatment costs and time.^143^ These technological innovations will provide new opportunities for the application of CAR‐T‐cell therapy in patients with GSRCC. Most importantly, the development of personalized treatment plans is another future research direction, such as selecting appropriate CAR‐T‐cell targets on the basis of the patient's molecular characteristics and immune status.

As an active immunotherapy method, tumor vaccines stimulate the body's own immune system to recognize and attack tumor cells.^144^, ^145^ However, to date, there have been no phase III clinical trials of tumor vaccines specifically targeting GSRCC. Most studies are in early stages (phase I/II) and are included within broader gastric cancer trials. Neoantigen vaccines are a personalized cancer immunotherapy that involve analyzing tumor‐specific mutations in patients to identify neoantigens and designing customized vaccines to precisely activate the immune system's attack on cancer cells. For example, a phase I trial (ChiCTR1800017319) evaluated the safety, immunogenicity, and preventive efficacy of a neoantigen vaccine in preventing tumor recurrence in high‐risk gastric/gastroesophageal junction cancer patients (including patients with GSRCC) after adjuvant chemotherapy for postsurgical resection.^146^ The results revealed 1‐ and 2‐year disease‐free survival (DFS) rates of 96.6% (28/29) and 82.4% (14/17), respectively, with clinical outcomes superior to the 17‐month DFS reported in previous studies for stage IIIB gastric cancer patients who underwent D2 radical gastrectomy followed by adjuvant chemotherapy and the 16‐month DFS for stage IIIC patients. Moreover, another clinical trial evaluating the safety and efficacy of personalized neoantigen vaccines for the treatment of advanced gastric cancer (including GSRCC) is currently underway (ClinicalTrials.gov identifier NCT05227378), and we look forward to good results in the future. Encouragingly, a case report explored the efficacy of a neoantigen‐loaded dendritic cell vaccine combined with anti‐PD‐1 therapy in advanced gastric cancer, and the results revealed that combination therapy triggered a stronger immune response and complete tumor regression.^147^ Although the positive rate of HER2 in GSRCC is low (<10%), peptide vaccines targeting HER2 may be suitable for specific subgroups (ClinicalTrials.gov identifier NCT05315830). Notably, mRNA vaccines, such as Moderna's mRNA‐4157/V940, leverage malignant cell‐specific neoantigens to amplify adaptive immune responses and have achieved success in areas such as melanoma.^148^ However, mRNA vaccines remain in the preclinical exploration stage for GSRCC, with the key challenge being how to identify highly immunogenic neoantigens from the limited number of mutations in GSRCC. In the future, with the development of personalized new antigen identification technology, new vaccine platforms, and combination strategies, vaccine treatment for GSRCC is expected to become a powerful means of overcoming current treatment bottlenecks.^149^, ^150^

Individual differences significantly affect the outcome of immunotherapy, and patient responses vary greatly. Even among those with the same pathological type of gastric cancer, the effectiveness of immunotherapy can differ greatly. This may be related to multiple factors, such as the patient's genetic background, immune status, and tumor microenvironment.^151^ How to overcome such individual differences is one of the challenges faced by tumor immunotherapy for treatment of GSRCC.

## 8 IMMUNOTHERAPY SENSITIZATION STRATEGY FOR GSRCC

Some chemotherapy drugs, such as oxaliplatin and paclitaxel, can induce immunogenic cell death in tumors, releasing a series of damage‐associated molecular patterns (DAMPs), ATP, and HMGB1, which act as messengers to attract and activate dendritic cells to initiate antitumor immunity.^152^ Phase III studies, such as CheckMate‐649 and ORIENT‐16, have established “chemotherapy (XELOX or SOX) + PD‐1 inhibitors (nivolumab/sintilimab)” as the first‐line standard treatment for advanced gastric cancer.^153^, ^154^ Although these studies did not focus specifically on the GSRCC subgroup, this regimen provides a fundamental sensitization framework for all pathological types, including GSRCC. Paclitaxel can remodel the tumor microenvironment through multiple pathways. For instance, it can influence the expression of various cytokines and adhesion molecules in the tumor microenvironment and promote the infiltration and activation of immune cells, thus transforming “cold” tumors into “hot” tumors and enhancing sensitivity to immunotherapy.^155^, ^156^ Nab‐paclitaxel, as a novel formulation, directly attacks tumor cells and recruits mast cells and proinflammatory macrophages to further optimize the immune microenvironment and improve the efficacy of combination therapy.^157^ Additionally, paclitaxel‐based chemotherapy may induce stromal damage and interfere with the dormant state of cancer cells, reactivating dormant tumor cells; this suggests its potential role in altering the physical barriers of the stroma and intercellular interactions.^158^ Therefore, paclitaxel serves as a crucial cornerstone in the GSRCC combination regimen. The POF regimen refers to a chemotherapy combination used to treat advanced gastric cancer, consisting of paclitaxel, oxaliplatin, and the fluorouracil/leucovorin (5‐FU/Leucovorin) backbone. The POF regimen has recently been proven safe and effective as a first‐line therapy for patients with advanced GSRCC.^159^ However, the potential of integrating the POF regimen with anti‐HER2 therapy and/or anti‐PD‐1/PD‐L1 immunotherapy in advanced GSRCC patients warrants further investigation.

The tumor microenvironment of GSRCC seems to be in a quiescent state, making it difficult for CD4^+^ and CD8^+^ T cells to be mobilized, thus impairing the normal function of B cells.^78^ The depletion of CXCL13 derived from CD8‐Tex cells and the absence of mature tertiary lymphoid structures are key reasons for the low response to immunotherapy in patients with GSRCC.^78^ CXCL13 expression may predict the response of patients with GSRCC to immune checkpoint blockade, possibly because of its effect on tertiary lymphoid structures. For non‐GSRCC patients, conventional treatments, including chemotherapy and/or immune checkpoint blockade, may be feasible, whereas for GSRCC patients, enhancing the ability of CD8‐Tex cells to produce CXCL13 may be key to improving the immune therapy response.^78^ Notably, GSRCC cells are rich in mitogen‐activated protein kinases and estrogen signaling pathways, which can positively reinforce each other.^78^ Therefore, targeting these pathways may have synergistic effects with immunotherapy, but further verification is needed.

Mogamulizumab is a monoclonal antibody that targets C‐C chemokine receptor 4 (CCR4), which is highly expressed by Treg cells.^160^ Mogamulizumab is often used for treating mycosis fungoides, relapsed/refractory adult T‐cell leukemia, cutaneous T‐cell lymphoma, and Sezary syndrome.^161^ Previous clinical trials investigating the use of mogamulizumab in hematological diseases have reported a reduction in Tregs in peripheral blood, indicating that mogamulizumab may facilitate the depletion of Tregs.^162^ Recently, a phase I trial (ClinicalTrials.gov identifier NCT02946671) evaluated the safety and efficacy of preoperative combination therapy with mogamulizumab (anti‐CCR4) and nivolumab (anti‐PD‐1) in patients with solid cancers. Among the clinical responses, three were partial responses (one lung and two esophageal cancers), and one was a complete response (lung cancer). Pathological responses included nine partial responses (five kidney, two lung, and two esophageal cancers). Treg cells were depleted in all tumors, and 50% of the patients had increased lymphocytes in tumor tissue, which correlated with a better prognosis.^163^ Although this clinical trial did not specifically target patients with GSRCC, it highlights directions for the future application of combination therapy with mogamulizumab and nivolumab in patients with GSRCC. As Tregs are substantially enriched in GSRCC, targeting these cells may effectively reverse the immunosuppressive microenvironment.^23^

Anti‐vascular drugs (such as ramucifumab and apatinib) can temporarily normalize distorted tumor blood vessels, improve blood flow and oxygen supply, and promote the infiltration of cytotoxic T cells. Apatinib, a tyrosine kinase inhibitor, can remodel the immunosuppressive tumor microenvironment by increasing CD8+ T‐cell and IGHA+ plasma cell infiltration while reducing the number of M2 macrophages.^164^ A randomized, multicenter, phase 3 clinical trial is currently being conducted to evaluate the efficacy and safety of XELOX combined with apatinib versus XELOX as postoperative chemotherapy in patients with locally advanced GSRCC (ClinicalTrials.gov identifier NCT03355612). Furthermore, many studies have shown that immunotherapy combined with apatinib has a synergistic effect on advanced gastric cancer (including GSRCC).^165^, ^166^

CLDN18.2 is linked to immune cell infiltration and PD‐L1 expression.^167^ CLDN18.2 is highly expressed in some GSRCCs.^38^ Phase III studies (such as GLOW) have shown that zolbetuximab (a CLDN18.2 monoclonal antibody) combined with chemotherapy significantly improves survival in CLDN18.2‐positive gastric cancer patients (including patients with GSRCC).^40^ In addition, a combination trial of zolbetuximab with other treatments in CLDN18.2‐positive gastroesophageal junction adenocarcinoma or gastric cancer patients (including GSRCC) is currently underway (ClinicalTrials.gov identifier NCT03505320). Participants in this study will receive one of the following treatments: zolbetuximab alone, zolbetuximab with chemotherapy, zolbetuximab with pembrolizumab, or zolbetuximab with chemotherapy and nivolumab. Although this study did not specifically target GSRCC, it represents a highly promising sensitization strategy for this disease. In summary, the sensitization of GSRCC to immunotherapy is a systematic project aimed at reversing its immunosuppressive microenvironment at multiple levels. With a more in‐depth understanding of the biological characteristics of GSRCC, more precise and efficient combination strategies are being developed.

However, the core limitation of current research on GSRCC is the lack of preclinical models capable of simulating its tumor immune microenvironment. Existing mainstream models, such as cell line‐derived xenografts (CDXs) and patient‐derived xenografts (PDXs) based on immunodeficient mice, fail to reconstruct a functional immune system, rendering a series of critical scientific questions unanswered.^168^ These include specific immune evasion mechanisms, resistance to immunotherapy, and complex interactions between the tumor stroma and immune cells. Although emerging approaches such as humanized mouse models and organoid‐immune cell coculture systems show potential, their application in this specific GSRCC subtype with low modeling success rates still faces significant technical challenges. Therefore, developing models that integrate the pathological features of signet ring cells with intact immune functionality is a priority for achieving therapeutic breakthroughs in this field.

## 9 LIMITATIONS OF CURRENT EVIDENCE

Despite significant advances in current research (Table 1), the evidence still presents several critical limitations that may affect the robustness and generalizability of the conclusions. First, many studies are limited by small sample sizes, which not only limit statistical power but also may lead to overreliance on specific populations or rare subgroups, compromising the reproducibility of the findings. Second, most conclusions have not been sufficiently validated in independent cohorts and lack external verification across multiple centers and diverse populations, introducing uncertainty regarding their biological significance and clinical translational potential. Additionally, studies relying on single‐cell sequencing technologies often face challenges from technical noise, batch effects, and variability in analytical pipelines, making comparisons and integration across different studies difficult. This may introduce variability in conclusions related to cell type identification, trajectory inference, and other aspects. Therefore, future research needs to enhance the reliability and generalizability of evidence by expanding sample sizes, conducting multicenter validation, and standardizing analytical approaches.

**TABLE 1:** Recently published single‐cell, genomic, and immunoprofiling studies on GSRCC.

	Sample size	Key findings	Datasets	Implications	Limitations
Single‐cell studies					
Zhao et al.^23^	In total, 13 GSRCC patients were enrolled for scRNA‐seq, including 7 males and 6 females who were 51 to 63 years old.	(1) Microseminoprotein‐beta (MSMB) serves as a marker gene for identifying moderately to poorly differentiated adenocarcinoma (M/PDA) and GSRCC. (2) GSRCC cells exhibit reduced adhesion, enhanced immune evasion, and an immunosuppressive microenvironment, possibly contributing to GSRCC's poor prognosis. (3) SRCCC cells are notably enriched in MAPK and estrogen signaling pathways.	Accession code GSA‐Human: HRA003647	Compared to M/PDA cells, GSRCC cells possess distinct cytological features and a unique immune microenvironment that may be advantageous for precise diagnosis and treatment.	The sample size is small with a lack of analytical methods for multiomics.
Chen et al.^78^	In total, 7 GSRCC patients were enrolled for scRNA‐seq, including 4 males and 3 females who were ages 51 to 82 years old.	Improving exhausted CD8+ T cells' ability to produce CXCL13 may be vital for reversing the refractory state in GSRCC patients. The number and intensity of immune cell interactions were significantly reduced in GSRCC. The number of tertiary lymphoid structures (TLSs) assembled from T and B cells in GSRCC was reduced.	Not available	This study offers a single‐cell adaptive immune profile of GSRCC, highlighting specific T‐ and B‐cell states' roles in nonresponsive tumor immune microenvironments (TIME) and identifying CXCL13 as a key coordinator of GSRCC TIME activation by influencing tertiary lymphoid structure maturation.	This study is mainly observational with a small sample size, requiring functional assays to better understand CXCL13's role in GSRCC immunosuppression.
Spatial metabolomics studies					
Shi et al.^47^	Two GSCRC patients and 2 gastric adenocarcinoma patients were enrolled for spatial metabolomics analysis, including 3 males and 1 females who were 55 to 73 years old.	Many lipidomic biomarkers correlated with GSRCC were identified, encompassing phosphatidylethanolamine N‐methyl, phosphatidylethanolamine, sphingomyelin, diacylglycerol, phosphatidic acid, and phosphatidylcholine.		Lipid reprogramming was a significant finding in the study of GSRCC.	Further validation is needed to determine whether these metabolites can serve as specific diagnostic markers for GSRCC. The role of these metabolites in the progression of GSRCC still needs further validation. Small sample sizes.
Genomic studies					
Gallanis et al.^169^	Twenty samples from human total gastrectomy specimens from germline *CDH1* variant carriers were collected for multiomic analysis.	Spatial transcriptomic analysis showed lower *CDH1* expression and higher expression of extracellular matrix remodeling in early, multifocal signet ring cell (SRC) lesions compared to nearby unaffected gastric epithelium. Single‐cell RNA sequencing showed that early SRC lesions did not have changes in common gastric cancer drivers (*TP53*, *ARID1A*, *KRAS*), whereas advanced SRC had somatic mutations in TP53 and ERBB3.	Not available	The distinct genomic and transcriptomic profiles of early SRC lesions compared to advanced SRC suggest that SRC lesions may be precancerous in patients with germline *CDH1* mutations.	Lack of validation in independent cohorts.
Kawakami et al.^170^	A 35‐year‐old Japanese man with advanced gastric cancer underwent comprehensive genome profiling, while his mother, who had GSRCC, underwent germline multigene panel testing.	A germline pathogenic variant in the *CTNNA1* gene, specifically the p.Q662* mutation, is associated with an elevated risk of hereditary diffuse gastric cancer. This pathogenic variant of *CTNNA1* (p.Q662*) could not be detected by common germline multigene panel testing.	Not available	A rare germline pathogenic variant of *CTNN1A* can be identified through comprehensive genomic profiling.	(2) Small sample sizes. (3) Lack of validation in independent cohorts.
Nasri et al.^171^	In this study, formalin‐fixed paraffin‐embedded (FFPE) specimens from 12 individuals belonging to a single GSRCC kindred were used.	In contrast to advanced diffuse gastric cancer (DGC), early intramucosal hereditary diffuse gastric cancer (eHDGC) is distinguished by reduced genomic instability at fragile sites. eHDGC exhibits an active DNA damage response, which offers a molecular explanation for the observed indolence of eHDGC.	Not available	This finding is crucial for understanding DGC's pathology and suggests a revision of the current definition of eHDGC as a malignant disease.	These observations, while informative, do not constitute definitive evidence.
Immunoprofiling studies					
Jin et al.^42^	In total, 89 GSRCC patients were enrolled, including 67 males and 22 females who were 32 to 81 years old.	A study of 89 patients with advanced GSRCC found that 40.4% of patients expressed PD‐L1 and 18.0% expressed PD‐1, with a significant correlation between the two expressions. Additionally, high CD3+ T‐cell infiltration was associated with better overall survival, suggesting that immune checkpoint inhibitors may have a potential role in the treatment of GSRCC.	Not available	CD3+ T‐cell infiltration and PD‐1/PD‐L1 expression serve as potential immunotherapy biomarkers in advanced GSRCC.	(2) Lack of validation in independent cohorts. (2) Small sample sizes.
Xu et al.^38^	In total, 105 formalin‐fixed, paraffin‐embedded (FFPE) tissue samples with advanced GSRCC histology were collected to test CLDN18.2 expression.	(1) Off the 105 advanced gastric GSRCC samples analyzed, 95.2% (100 samples) showed positive staining, and 64.8% (68 samples) exhibited moderate‐to‐strong CLDN18.2 expression. (2) CLDN18.2 expression might be related to GRIN2A mutation in GSRCC.	Not available	The high expression of CLDN18.2 in advanced gastric GSRCC patients confirms the value of targeting CLDN18.2 in this type of cancer.	(1) Lack of validation in independent cohorts. (2) Small sample sizes.
Green et al.^101^	In total, 20 GSRCC patients were enrolled, including 6 males and 14 females who were 20 to 65 years old.	The increase in Treg cells in early GSRCC is a potential initiating step for immune evasion. The elevation of CD4+ T cells in early GSRCC lesions is associated with clinically observed GSRCC dormancy.	The datasets generated in this study are included in Data S1.	The upregulation of Tregs may constitute a potential mechanism for immune evasion.	(1) The RNA yields from four samples in this study are below the critical value for analysis. (2) The study found a correlation but did not establish a clear mechanistic link between tumor dormancy and immune cell subsets.
Hirotsu et al.^172^	In total, 38 formalin‐fixed, paraffin‐embedded (FFPE) tissue samples with GSRC histology were collected to test mismatch repair (MMR) proteins expression.	All GSRCC tumors (*n* = 38) normally expressed MMR proteins (MLH1, MSH2, MSH6 and PMS2 proteins), whereas 22 of 109 of NSRC samples (20%) showed deficient MMR proteins. Analysis of the Cancer Genome Atlas (TCGA) database revealed that deficient MMR was present in only 6 of 99 (6%) GSRCC tumor samples, compared to 64 of 304 (21%) in intestinal gastric tumor samples.	The datasets are included in this study.	The deficiency of mismatch repair genes was observed less frequently in GSRCC compared to non‐GSRCC.	(1) Lack of validation in independent cohorts. (2) Small sample sizes.

## 10 SUMMARY AND FUTURE PROSPECTS

Future research on GSRCC will pursue multiple avenues (Figure S2). With regard to molecular mechanisms, it is imperative to conduct an in‐depth investigation into how gene mutations, such as *CDH1* in hereditary diffuse gastric cancer, facilitate the progression of tumors from early indolent lesions to aggressive disease states. Elucidating the associated molecular events and regulatory networks will contribute to the development of more precise therapeutic targets.^173^, ^174^ Furthermore, in‐depth research on the relationships between molecular markers such as CLDN18.2 and other gene variations will aid in the development of more effective targeted therapeutic drugs and combination therapy regimens.^38^, ^175^ In addition, by integrating multiomics technologies such as single‐cell sequencing and spatial metabolomics, it is helpful to comprehensively analyze the characteristics and dynamic changes in the microenvironment of GSRCC, providing a more solid foundation for the development of novel treatment strategies.

## AUTHOR CONTRIBUTIONS

**Qian Wang:** Writing – original draft; conceptualization; writing – review and editing. **Shuai Zhou:** Writing – original draft; methodology. **Xiongchao Fang:** Software; validation; formal analysis. **Xianli He:** Project administration; supervision. **Gang Wang:** Investigation; funding acquisition. **Nan Wang:** Writing – review and editing; resources; supervision; conceptualization.

## CONFLICT OF INTEREST STATEMENT

The authors declare no conflict of interest.

## Supporting information


**Figure S1.** The treatment strategy for GSRC. Current treatment methods for GSRC include immunotherapy, targeted therapy, endoscopic therapy, surgical treatment, chemotherapy and etc. GSRC, gastric signet ring cell carcinoma; TEFOX regimen, docetaxel‐5FU‐oxaliplatin.
**Figure S2.** Research direction of GSRC. Future research on GSRC will explore multiple aspects, including the early diagnosis, metabolic characteristics, precise molecular typing, targeted therapy, and immunotherapy of GSRC. GSRC, gastric signet ring cell carcinoma; PDO, patient‐derived organoid; PDX, patient‐derived tumor xenograft; EBV, Epstein–Barr virus.
