# Supplementary material for: Immune, molecular and genetic profiles of gastric signet ring cell carcinoma: Recent progress and future challenges
Source: Int J Cancer. 2026 Mar 27;159(1):11–29. doi: 10.1002/ijc.70416 (PMC13139998; doi:10.1002/ijc.70416)
Supplement: Supplementary file 1 — Figure S1. The treatment strategy for GSRC. Current treatment methods for GSRC include immunotherapy, targeted therapy, endoscopic therapy, surgical treatment, chemotherapy and etc. GSRC, gastric signet ring cell carcinoma; TEFOX regimen, docetaxel‐5FU‐oxaliplatin. Figure S2. Research direction of GSRC. Future research on GSRC will explore multiple aspects, including the early diagnosis, metabolic characteristics, precise molecular typing, targeted therapy, and immunotherapy of GSRC. GSRC, gastric signet ring cell carcinoma; PDO, patient‐derived organoid; PDX, patient‐derived tumor xenograft; EBV, Epstein–Barr virus. [file IJC-159-11-s001.pdf]

The immune, molecular and genetic profiles of gastric signet ring cell carcinoma: recent progress and future challenges

Qian Wang, Shuai Zhou, Xiongchao Fang, Xianli He, Gang Wang, Nan Wang

Table of contents

Supplementary figures

Fig. S1...2

Fig. S2...3

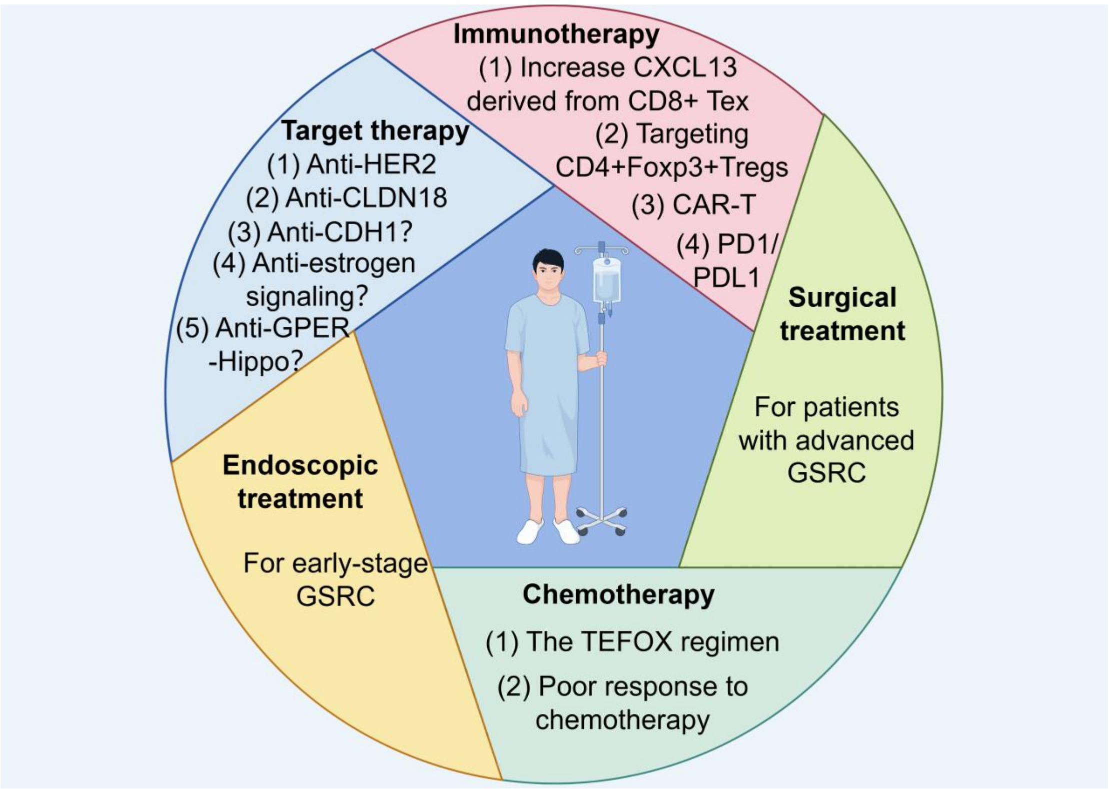

Supplementary figure 1. The treatment strategy for GSRC. Current treatment methods for GSRC include immunotherapy, targeted therapy, endoscopic therapy, surgical treatment, chemotherapy and etc. Abbreviation: GSRC, gastric signet ring cell carcinoma; TEFOX regimen, docetaxel-5FU-oxaliplatin.

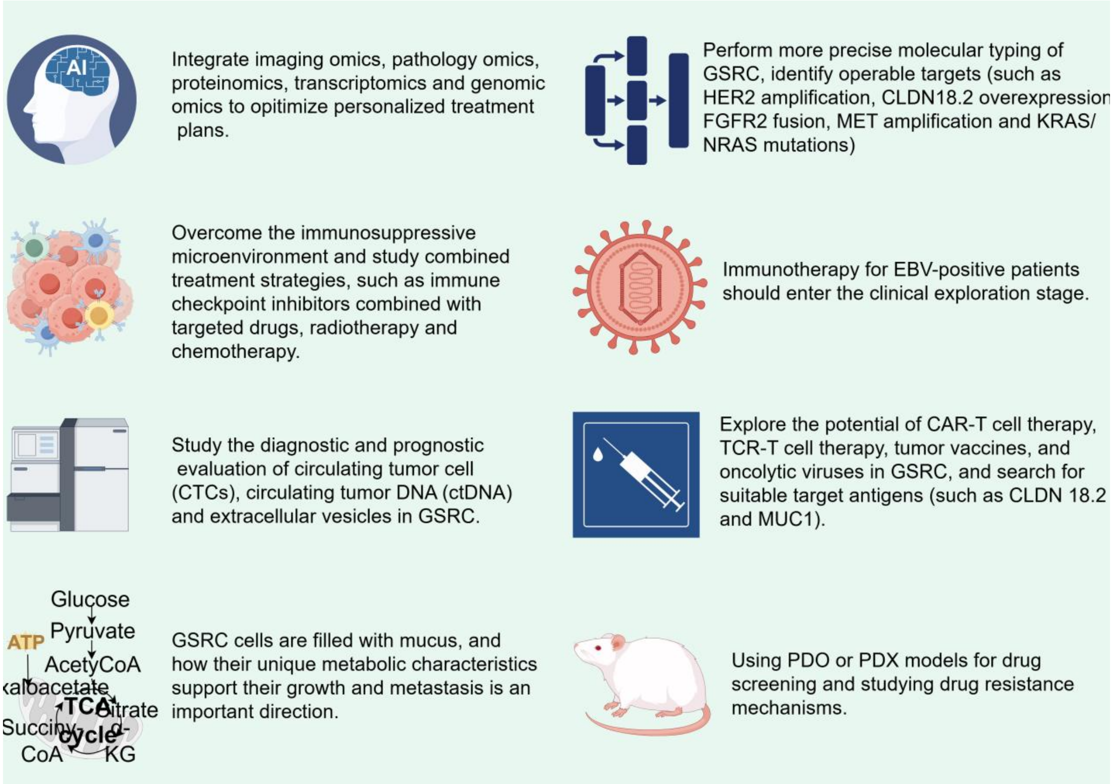

Supplementary figure 2. Research direction of GSRC. Future research on GSRC will explore multiple aspects, including the early diagnosis, metabolic characteristics, precise molecular typing, targeted therapy, and immunotherapy of GSRC. Abbreviation: GSRC, gastric signet ring cell carcinoma; PDO; patient-derived organoid; PDX, patient-derived tumor xenograft; EBV, Epstein-Barr virus.
